# Identification of aging-related biomarkers and immune infiltration characteristics in osteoarthritis based on bioinformatics analysis and machine learning

**DOI:** 10.3389/fimmu.2023.1168780

**Published:** 2023-07-12

**Authors:** JiangFei Zhou, Jian Huang, ZhiWu Li, QiHe Song, ZhenYu Yang, Lu Wang, QingQi Meng

**Affiliations:** ^1^ Department of Orthopedics, Guangzhou Red Cross Hospital of Jinan University, Guangzhou, China; ^2^ Department of Traumatic Orthopaedics, The Central Hospital of Xiaogan, Xiaogan, Hubei, China; ^3^ Department of Orthopedics, The 2nd People’s Hospital of Bijie, Bijie, Guizhou, China; ^4^ Department of Neurology, The Central Hospital of Xiaogan, Xiaogan, Hubei, China; ^5^ Guangzhou Institute of Traumatic Surgery, Guangzhou Red Cross Hospital of Jinan University, Guangzhou, China

**Keywords:** osteoarthritis, aging-related genes, immune infiltration, WGCNA, machine learning, biomarkers

## Abstract

**Background:**

Osteoarthritis (OA) is a degenerative disease closely related to aging. Nevertheless, the role and mechanisms of aging in osteoarthritis remain unclear. This study aims to identify potential aging-related biomarkers in OA and to explore the role and mechanisms of aging-related genes and the immune microenvironment in OA synovial tissue.

**Methods:**

Normal and OA synovial gene expression profile microarrays were obtained from the Gene Expression Omnibus (GEO) database and aging-related genes (ARGs) from the Human Aging Genomic Resources database (HAGR). Gene Ontology (GO), Kyoto Encyclopedia of Genes and Genomes (KEGG), Disease Ontology (DO), and Gene set variation analysis (GSVA) enrichment analysis were used to uncover the underlying mechanisms. To identify Hub ARDEGs with highly correlated OA features (Hub OA-ARDEGs), Weighted Gene Co-expression Network Analysis (WGCNA) and machine learning methods were used. Furthermore, we created diagnostic nomograms and receiver operating characteristic curves (ROC) to assess Hub OA-ARDEGs’ ability to diagnose OA and predict which miRNAs and TFs they might act on. The Single sample gene set enrichment analysis (ssGSEA) algorithm was applied to look at the immune infiltration characteristics of OA and their relationship with Hub OA-ARDEGs.

**Results:**

We discovered 87 ARDEGs in normal and OA synovium samples. According to functional enrichment, ARDEGs are primarily associated with inflammatory regulation, cellular stress response, cell cycle regulation, and transcriptional regulation. Hub OA-ARDEGs with excellent OA diagnostic ability were identified as MCL1, SIK1, JUND, NFKBIA, and JUN. Wilcox test showed that Hub OA-ARDEGs were all significantly downregulated in OA and were validated in the validation set and by qRT-PCR. Using the ssGSEA algorithm, we discovered that 15 types of immune cell infiltration and six types of immune cell activation were significantly increased in OA synovial samples and well correlated with Hub OA-ARDEGs.

**Conclusion:**

Synovial aging may promote the progression of OA by inducing immune inflammation. MCL1, SIK1, JUND, NFKBIA, and JUN can be used as novel diagnostic biomolecular markers and potential therapeutic targets for OA.

## 1 Introduction

Age is the paramount risk factor for osteoarthritis (OA), which is one of the most common causes of chronic pain and disability in the elderly. The prevalence of OA is rising due to an increase in the number of elderly and obese people, negatively impacting patients’ quality of life and imposing a massive burden on families and society (1, 2). The pathogenesis of OA is extremely complex, involving mechanical overload, an increase in inflammatory mediators, metabolic changes and cellular senescence, all of which can interact to promote the development of OA (3). As a result, studying the molecular biology of OA and looking for potential biomarkers of OA is critical for early diagnosis and treatment of OA.

Aging is a complicated biological process. Senescent cells continue to accumulate in the human body as we age, leading to a variety of age-related diseases such as osteoarthritis, cardiovascular disease, Alzheimer’s disease, and so on (4–6). Cellular senescence is one of the first signs of aging, characterized by permanent cell cycle arrest and the release of harmful pro-inflammatory factors into the surrounding microenvironment, a feature known as Senescence-associated secretory phenotype, SASP (7). The persistence of SASP factors causes chronic, low-grade systemic inflammation, stimulates senescence in neighboring cells and accelerates aging progression (8). SASPs such as IL-1, IL-6, MMP-13, VEGF and other pro-inflammatory factors have been found in OA cartilage and synovial fluid (9). Many synovial fibroblasts positive for p16 and SA-β-Gal have been found in the synovium of elderly and OA patients. Senescent synovial fibroblasts can aggravate synovial inflammation and cause cartilage degradation by secreting pro-inflammatory factors and matrix metalloproteinases (10). Intriguingly, injection of senescent fibroblasts into the knee joint cavity of mice resulted in inflammation, cartilage erosion and osteophyte formation (11). In post-traumatic mouse models, targeted removal of senescent cells in mouse knee articular cartilage and synovium specifically marked by P16 can effectively reduce the development of inflammatory response and articular cartilage injury-related pain (12). Aging is clearly involved in multiple pathways of OA pathogenesis, but the precise mechanism remains unknown.

**Figure 1:**
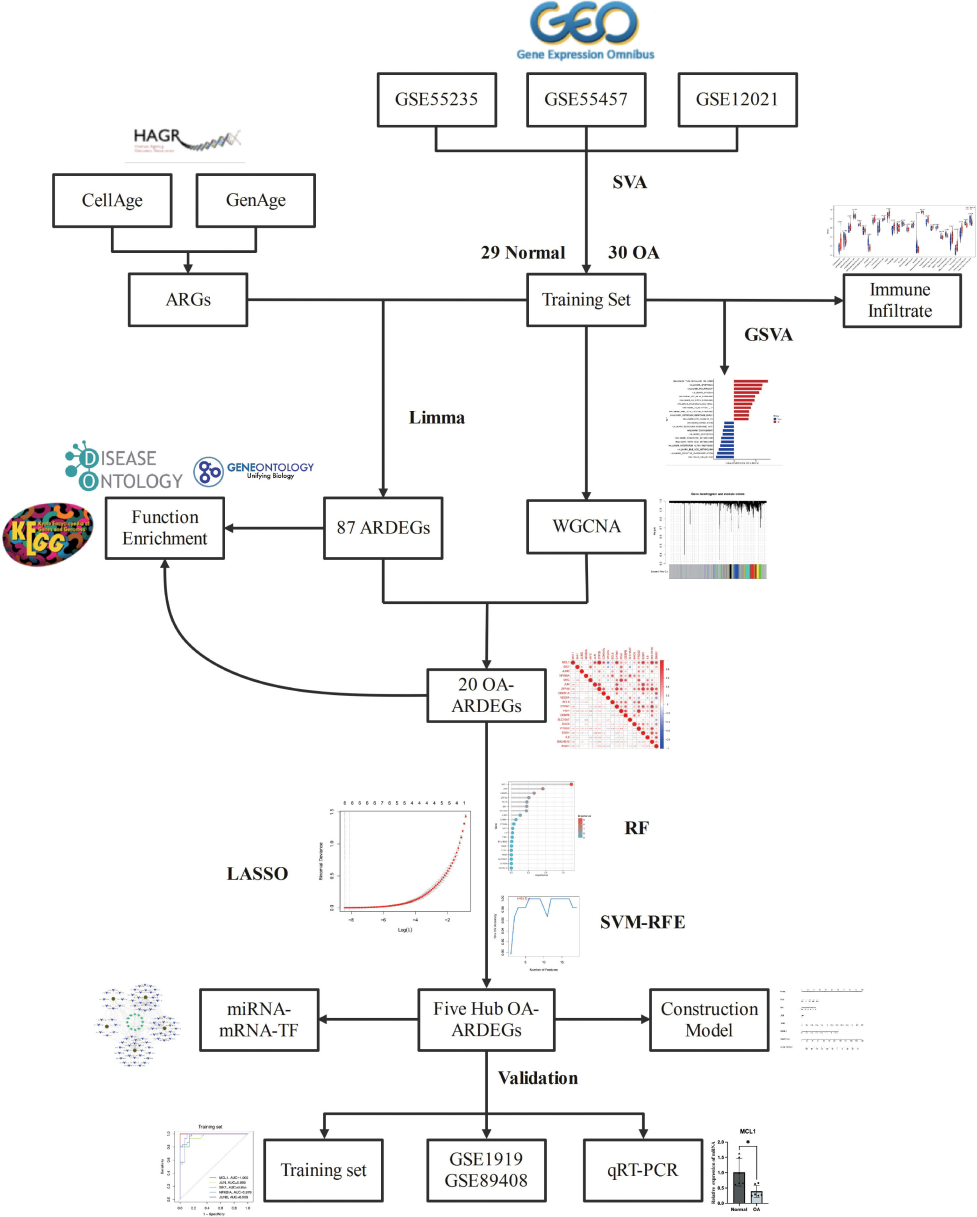
Flowchart for Comprehensive Analysis of Aging-Related Genes in Osteoarthritis.

Bioinformatics is a multidisciplinary field. With the development of high-throughput sequencing technology and the application of machine learning in the medical field in recent years, new ideas for studying the molecular mechanisms of various diseases have emerged (13, 14). WGCNA and three machine learning algorithms were applied in this study to screen the Hub OA-ARDEGs of OA and an online database to predict their potential miRNAs and TFs. The ssGSEA algorithm was often used to investigate OA’s immune infiltration profile and its relationship with Hub OA-ARDEGs. It provides a new direction for the early diagnosis and treatment of OA. The overall workflow of this study is depicted in **Figure 1**.

## 2 Materials and methods

### 2.1 Gene expression dataset screening and processing

We downloaded the gene expression profile microarrays (GSE55235, GSE55457, GSE12021, GSE1919, GSE89408) for normal and OA synovial samples from the Gene Expression Omnibus (GEO), with the information shown in **Table 1**. Probe names were converted to gene names using Perl and with the help of platform annotation files. Background correction and normalization of each dataset using the R package limma and integrating three synovial datasets from the same platform using the R package sva to remove batch effects (15). Two-dimensional PCA clustering plots were used to show the before and after differences in removing batch effects from the samples. For subsequent analysis, the merged dataset served as the training set and the GSE1919 and GSE89408 dataset served as the validation set.

**Table 1:** Descriptive statistics.

GEO (ID)	Platform	Tissue (Homo sapiens)	Samples (number)		Attribute
			Normal	OA	
GSE55235	GPL96	Synovium	10	10	Training
GSE55457	GPL96	Synovium	10	10	Training
GSE12021	GPL96	Synovium	9	10	Training
GSE1919	GPL91	Synovium	5	5	Validation
GSE89408	GPL11154	Synovium	28	22	Validation

GPL96 (Affymetrix Human Genome U133A Array). GPL91(Affymetrix Human Genome U95A Array). GPL11154 (Illumina HiSeq 2000 (Homo sapiens).

### 2.2 Download and collation of aging-related genes

Human aging-related genes (ARGs), including GenAge (307) and CellAge (279), were obtained from the Human Aging Genomic Resources (HAGR, https://genomics.senescence.info/) database (16). After merging and deleting duplicate genes, 543 ARGs were obtained for subsequent analysis. The list of aging-related genes is detailed in **Table S1** of the **Supplementary Material**.

### 2.3 Identification of aging-related differentially expressed genes

ARGs expression matrices were extracted from the training set and analyzed for differences using the R package limma, with |logFC|>0.5; FDR<0.05 as the criterion for screening to obtain aging-related differential genes (ARDEGs) in normal and OA synovial samples (17). A heat map of gene expression was created to visualize the top 30 genes with the most significant differences.

### 2.4 Construction of protein-protein interaction networks network

To evaluate the gene interactions among the ARDEGs, a protein-protein interaction (PPI) network was constructed using the Search Tool for the Retrieval of Interacting Genes (STRING, https://cn.string-db.org/) database (18), with a confidence score of >0.7 as the cut-off criterion.

### 2.5 Functional enrichment analysis

R package Clusterprofiler and DOSE were used to perform Gene Ontology (GO), Kyoto Encyclopedia of Genes and Genomes (KEGG), and Disease Ontology (DO) enrichment analysis of ARDEGs, with q-value<0.05 set as a screening criterion to investigate their biological functions, signaling pathway enrichment, and disease similarities.

### 2.6 Gene set variation analysis

Gene set variation analysis (GSVA) is an unsupervised, non-parametric method for assessing transcriptome gene set enrichment (19). We used the gene sets “Hallmark.all.v2022.1.Hs.symbols” and “ c2.cp.kegg.symbols “ from the Molecular Signature Database (MSigDB) as reference sets. To assess the enrichment of normal and OA synovium samples, the R package GSVA was used to score the HALLMARKS and KEGG pathways. Significant enrichment was defined as FDR<0.05.

### 2.7 WGCNA and screening for ARDEGs with highly correlated OA features

Weighted Gene Co-expression Network Analysis (WGCNA) is an algorithm that finds biologically significant co-expressed gene modules and explores the relationship between gene networks and disease (20). We used the R package WGCNA to build a weighted co-expression network on the training set, analyzing the genes with the highest 50% expression variance in all expression profiles. The goodSamplesGenes function was used to check for missing values in the data, and the “pickSoftThreshold” function was used to filter and validate the best soft threshold. The Pearson correlation coefficient was used to create the adjacency matrix, which was then transformed into a topological overlap matrix (TOM) with appropriate power values and phase anisotropy (1-TOM). TOM classified the genes into different modules. The genes most associated with OA traits in the module were selected as cor.MM>0.7 and cor.GS>0.5. 0.5 is the screening condition for Hub genes in the module, and the intersection of Hub genes and ARDEGs in the module is taken to obtain OA-ARDEGs. The Pearson correlation test assessed the interaction of OA-ARDEGs at the mRNA level.

### 2.8 Identification of Hub ARDEGs with highly correlated OA features

LASSO regression, SVM-RFE, and random forest are methods for screening and identifying Hub OA-ARDEGs. The least absolute shrinkage and selection operator (LASSO) regression is a common data mining method (21). R package glmnet was applied to incorporate OA-ARDEGs into the diagnostic model, set the α value of the glmnet function to 1, obtained the best λ by ten cross-validations finally obtained the Aging signature genes based on the best λ. Support vector machine recursive feature elimination (SVM-RFE) is a common machine learning method based on embedded methods (22). The R package e1071 helped us find the best variables and remove the feature vectors generated by the SVM, thus obtaining the Aging signature genes. Recursive Feature Elimination (RFE) of the Random Forest algorithm is a supervised machine learning method (23). Aging signature genes were identified by relative importance greater than one when the decision tree was set to 500. The intersection of the three machine-learning filtered Aging signature genes was defined as Hub OA-ARDEGs using R package Venn. The GSE1919 and GSE89408 dataset could validate the receiver operating characteristic curve (ROC) analysis of the diagnostic value of Hub OA-ARDEGs for OA.

### 2.9 Patients samples

The synovial tissue from six patients who underwent total knee replacement surgery for osteoarthritis (OA) and normal synovial tissue from six patients with meniscus injuries were obtained for this study. All patients signed informed consent forms, and the collection, processing, and analysis of the samples were conducted under the guidance of the Ethics Committee of the Guangzhou Red Cross Hospital, affiliated with Jinan University (Ethics Approval No. 2023-001-01).

### 2.10 qRT-PCR

The total synovial tissue RNA was extracted using Trizol (Servicebio), followed by reverse transcription of the total RNA into complementary DNA (cDNA) using Takara Prime Script^®^ RT Master Mix. Quantitative real-time polymerase chain reaction (qRT-PCR) was conducted using 2× SYBR Green qPCR Hub Mix (without ROX) (Service). The primer sequences for the Hub osteoarthritis-associated differentially expressed genes (Hub OA-ARDEGs) can be found in **Table S16** of the **Supplementary Material**. The GAPDH gene was utilized as an internal reference gene. Each biological sample was subjected to three technical replicates.

### 2.11 Construction of Hub OA-ARDEGs risk prediction model

To improve clinical applicability, we use the R package rms to create a nomogram with Hub OA-ARDEGs, where “Points” represents the score of candidate genes and “Total Points” represents the sum of the scores of all the genes listed above. The accuracy of the nomogram model was determined by calibration, clinical decision, and Clinical impact curve.

### 2.12 miRNA-TF-mRNA regulatory network of Hub OA-ARDEGs

To improve prediction accuracy, we used the miRTarBase (24), Starbase (25), and Targetscan (26) databases to predict the miRNAs of Hub OA-ARDEGs. The Enrichr database (http://amp.pharm.mssm.edu/Enrichr/) was also applied to predict the transcription factors (TF) of Hub OA-ARDEGs, with a p-value of 0.05 as a screening condition. Construction of miRNA-TF-mRNA regulatory networks and visualization of the networks using Cytoscape (3.9.1).

### 2.13 Immunological characteristics of OA

Single sample gene set enrichment analysis (ssGSEA), an extension of the GSEA method, is widely used in bioinformatics studies related to immune infiltration (27). We calculated enrichment scores for normal and OA synovial samples in 28 immune cells and 13 immune functions using the R package GSVA and visualized the results using the R package vioplot. Spearman correlation analysis was used to correlate Hub OA-ARDEGs with immune cells and immune function.

### 2.14 Statistical analysis

All statistical analyzes were performed in R (version 4.2.2). Comparisons among two groups were made using Wilcox test. Spearman’s correlation analysis was used to understand the relationship between the expression levels of Hub OA-ARDEGs and immune cells and immune function. Differences were deemed statistically significant where P < 0.05. Statistical analyses of qRT-PCR were presented as the mean ± standard deviation for at least three individual experiments, and the statistical significance of differences was determined with the unpaired, two-tailed Student t-test. (*P < 0.05; **P < 0.01; ***P < 0.001). P < 0.05 was considered as statistically significant.

## 3 Results

### 3.1 GEO data processing

We integrated three synovial datasets, GSE55235, GSE55457, and GSE12021, containing a total of 29 normal synovial and 30 OA synovial samples. As is shown in **Figure 1** of the **Supplementary Material**, the gene expression level and principal component analysis (PCA) of each sample before and after eliminating the batch effect.

### 3.2 Identification and PPI analysis of ARDEGs

**Figure 2:**
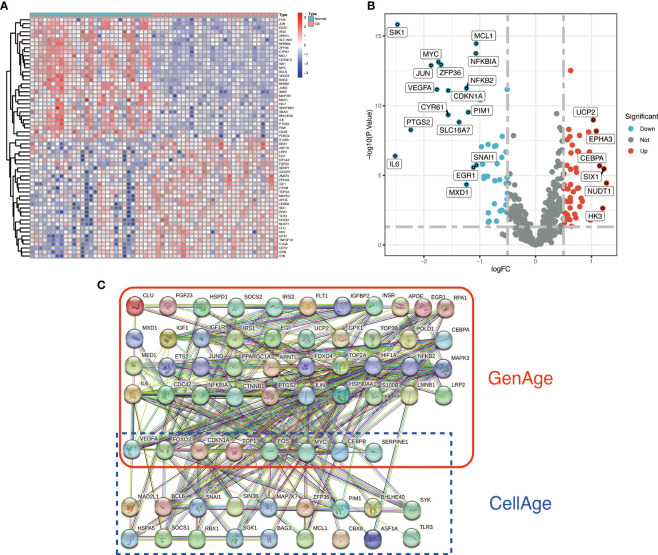
Identification and PPI analysis of ARDEGs. **(A)** heat map of the first 30 ARDEGs, with the left half representing normal synovial samples, the right half representing OA synovial samples, red representing upregulation and blue representing downregulation. **(B)** ARDEGs volcano plot. Red nodes indicate upregulated DEGs, blue nodes indicate downregulated DEGs, and grey nodes indicate genes that are not significantly differentially expressed. **(C)** Interaction map of 87 ARDEGs PPI protein networks.

We identified 87 ARDEGs using the R package limma and screening criteria of |logFC|>0.5 and FDR<0.05, of which 32 genes were upregulated in OA and 55 genes were downregulated in OA. **Table S2** of the **Supplementary Material** contains a detailed list of differentially expressed Aging-related genes. The heat map and volcano map were used to depict the differences (**Figures 2A, B**). PPI protein network interaction analysis revealed that ARDEGs interact closely at the protein level (**Figure 2C**). The results of the PPI protein network interaction analysis are available in the **Supplementary Material: Table S3**. 

### 3.3 Functional enrichment analysis of ARDEGs

**Figure 3:**
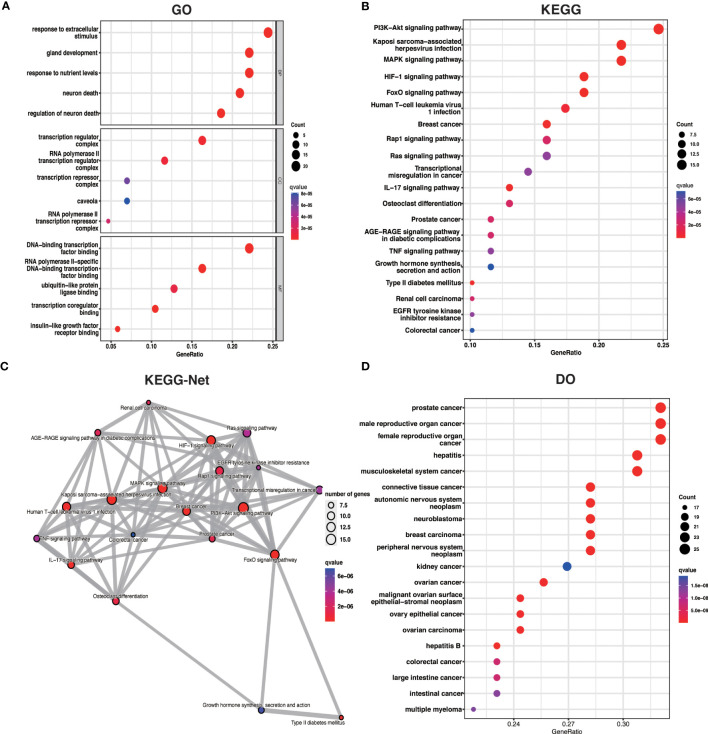
ARDEG functional enrichment analysis. **(A)** GO enrichment analysis with BP, CC, and MF included. The bubble plots depict the five most significantly enriched functions, where the size of the bubbles represents the number of DEGs (the larger the circle, the greater the number of DEGs) and the color represents the corrected p-value (the redder the color, the smaller the corrected p-value). **(B)** Analysis of KEGG enrichment, with bubble plots displaying the top 20 most significant pathway enrichments. **(C)** Map of KEGG pathway interactions. **(D)** DO enrichment analysis is depicted as a bubble diagram.

To better understand the potential mechanisms of ARDEGs in OA, we performed GO, KEGG, and DO enrichment analysis on ARDEGs using the R package clusterProfiler. GO enrichment analysis revealed that the first five ARDEG enrichments were primarily involved in the response to extracellular stimulus, neuron death, gland development, response to nutrient levels, and regulation of neuron death. The top 5 enriched items in Cellular Components (CC) and Molecular Functions (MF) are shown in **Figure 3A**. Furthermore, KEGG pathway analysis revealed that these ARDEGs were enriched in the HIF-1 signaling pathway, the FoxO signaling pathway, Kaposi sarcoma-associated herpesvirus infection, the PI3K-Akt signaling pathway, and the MAPK signaling pathway, and the pathways interacted closely (**Figures 3B, C**). DO enrichment analysis reveals disease types with similar pathogenic mechanisms of ARDEGs in OA, such as prostate cancer, male reproductive organ cancer, hepatitis B, female reproductive organ cancer, and hepatitis C (**Figure 3D**). **Tables S4–6** show detailed results for GO, KEGG, and DO enrichment of ARDEGs.

### 3.4 GSVA enrichment analysis

**Figure 4:**
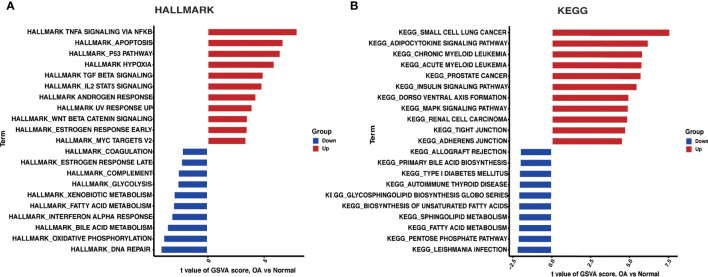
GSVA. **(A)** Differences in HALLMARKS pathway enrichment between OA and control groups. **(B)** Differences in KEGG pathway enrichment between OA and control groups.

We investigated HALLMARKS and KEGG pathway enrichment in OA through the GSVA method. TNFA signaling *via* the NFKB pathway, apoptosis, and the P53 pathway were significantly upregulated in OA when compared to the control group, according to HALLMARKS pathway enrichment results. At the same time, DNA repair, oxidative phosphorylation, and bile acid metabolism were all significantly reduced (**Figure 4A**). As is shown in the KEGG pathway enrichment results, the first three pathways were significantly upregulated in small cell lung cancer, adipocytokine signaling pathway, and chronic myeloid leukemia in OA. In contrast, leishmania infection, the pentose phosphate pathway, and fatty acid metabolism were all significantly downregulated (**Figure 4B**).

### 3.5 WGCNA

**Figure 5:**
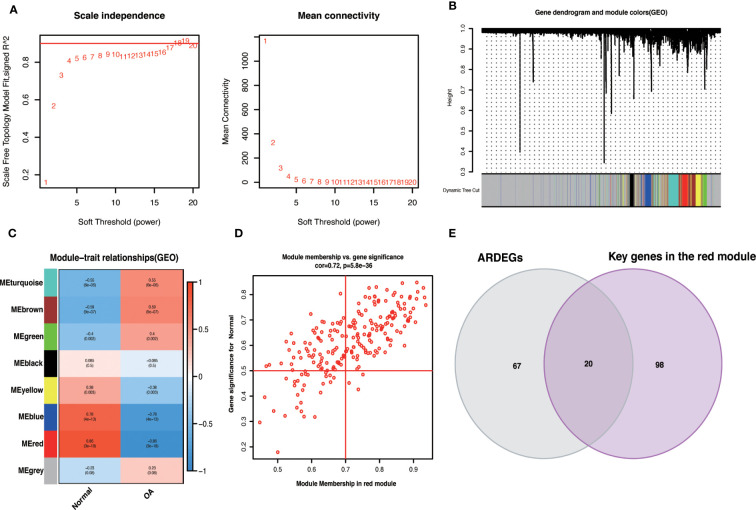
WGCNA. **(A)** Determine the best soft threshold. The soft threshold value of 18 was determined as the optimal choice for constructing a scale-free network based on the position of the red line (R^2 =^ 0.9). **(B)** The variance is in the top 50% of the gene cluster dendrogram, with each branch of the graph representing a gene and each color below representing a co-expression module. **(C)** Heat map of module-trait relationships, where each color represents a co-expression module and the values represent module-trait correlation coefficients and p-values. It can be seen that the red module has the highest correlation with OA. **(D)** Scatterplot of correlations between gene significance (GS) and module membership (MM) in red modules. **(E)** Venn diagram of the intersection of ARDEGs and Key genes in the red module.

We used the R package WGCNA, expression variance in the first 50% of genes as a screening condition to eliminate less volatile genes, and 6538 genes for co-expression network construction. The value 18 was selected as the optimal soft threshold (R^2 =^ 0.9) to establish a scale-free network (**Figure 5A**). Following that, cluster analysis was used to identify highly similar modules, with the minimum module size set at 60. Using dynamic hybridization shearing, eight gene modules were obtained, with one red module (217 genes) having the highest correlation (cor) with OA (cor = 0.86; P = 3e-18) (**Figure 5C**). Furthermore, there was a strong correlation between GS and MM within the red module (cor = 0.72; P=5.8e-36) (**Figure 5D**). The genes in the red module with cor.MM>0.7 and cor.GS>0.5 were chosen as the Hub genes and intersected with ARDEGs to yield 20 with OA-ARDEGs (**Figure 5E**). The results of co-expression modules for datasets can be found in the **Supplementary Material: Table S7**.

### 3.6 Correlation and enrichment analysis of OA-ARDEGs

**Figure 6:**
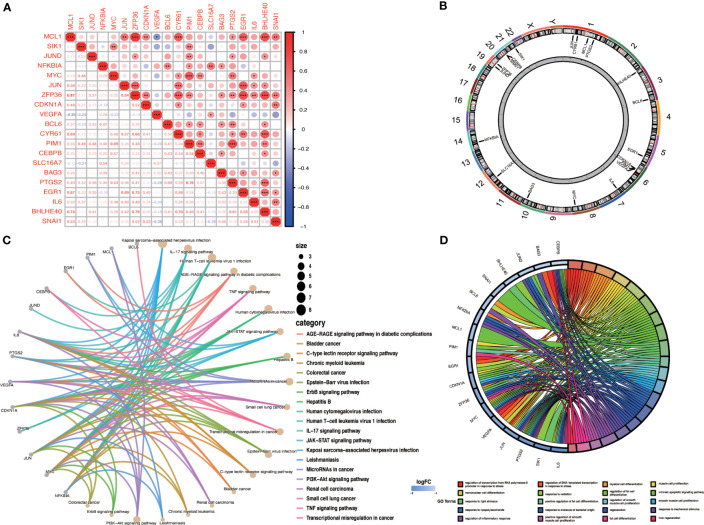
Correlation and enrichment analysis of OA-ARDEGs. **(A)** Correlation analysis of OA-ARDEGs, *P<0.05, **P<0.01, ***P<0.001. **(B)** Chromosome distribution of the 20 OA-ARDEGs. **(C)** KEGG enrichment analysis circle diagram. **(D)** Chord diagram of the top 20 GO entries for OA-ARDEGs GO enrichment.

We evaluated the correlation between OA-ARDEGs by the Pearson correlation coefficient. MCL1 was found to be highly correlated with ZFP36 (cor=0.87) and BHLHE40 (cor=0.74) (**Figure 6A**). **Figure 6B** depicts the chromosomal location of OA-ARDEGs. **Supplementary Material: Table S8**. KEGG pathway enrichment analysis revealed that the first five pathways enriched by OA-ARDEGs were primarily involved in Kaposi sarcoma-associated herpesvirus infection, IL-17 signaling pathway, Human Tcell leukemia virus 1 infection, AGER-AGE signaling pathway in diabetic complications, and TNF signaling pathway (**Figure 6C**). GO enrichment analysis shows that OA-ARDEGs are enriched in biological processes such as regulation of transcription from RNA polymerase II promoter in response to stress, regulation of DNA-templated transcription in response to stress, myeloid cell (**Figure 6D**). The specific results of KEGG and GO enrichment were shown in **Supplementary Material: Table S9, 10**.

### 3.7 Identification and validation of Hub OA-ARDEGs

**Figure 7:**
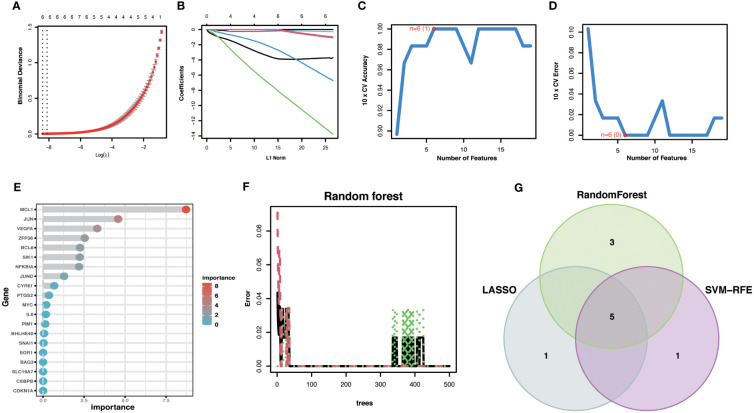
Machine Learning Screening Hub OA-ARDEGs. **(A)** LASSO coefficient analysis. Vertical dashed lines are plotted at the best lambda. **(B)** Ten cross-validations of the choice of adjustment parameters in the LASSO model. Each curve corresponds to one gene. **(C, D)** Maximum accuracy and minimum error plots of the SVM-RFE algorithm for screening optimal OA-ARDEGs. **(E)** Ranking of the relative importance of OA-ARDEGs. **(F)** Relationship between the number of random forest trees and the error rate. **(G)** LASSO, Random Forest, and SVM-RFE algorithms for screening Venn diagrams of the intersection of Aging signature genes.

To improve the accuracy of Hub OA-ARDEGs diagnostic OA, we used three machine learning algorithms, LASSO (**Figures 7A, B**), SVM-RFE (**Figures 7C, D**), and Random Forest (**Figures 7E, F**), to screen OA-ARDEGs. After combining the results of the three algorithms, a total of five Hub OA-ARDEGs were obtained, namely, MCL1, SIK1, JUND, NFKBIA, and JUN (**Figure 7G**). Results of three machine learning identification Hub OA-ARDEGs are shown in the **Supplementary Material: Table S11**.

### 3.8 Construction of Hub OA-ARDEGs risk prediction model

**Figure 8:**
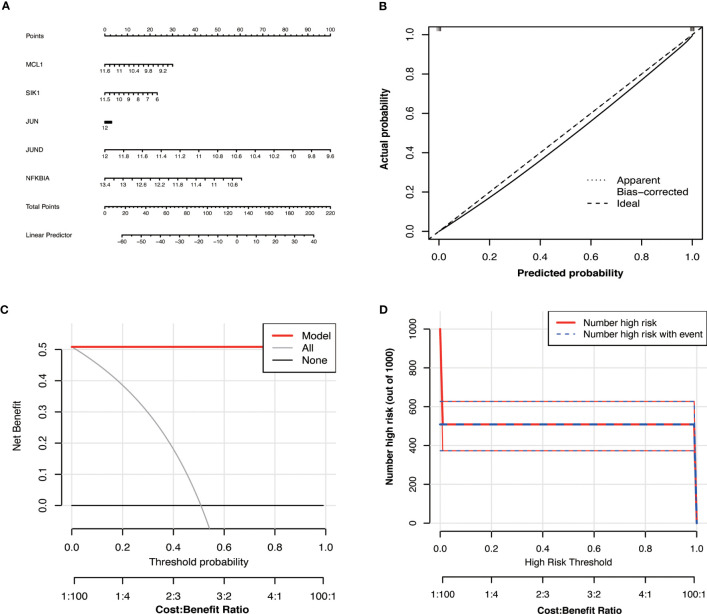
Hub OA-ARDEGs risk prediction model. **(A)** Nomogram of Hub OA-ARDEGs in the diagnosis of OA patients. **(B)** Calibration curve used to estimate the predictive accuracy of the nomogram (the closer to the ideal dashed line, the more reliable the result). **(C)** Accuracy of the clinical decision curve detection model (the further the red line endpoints are from the grey line, the higher the accuracy). **(D)** Clinical impact curve (The solid red line indicates the number of people identified by the model as being at high risk for different probability thresholds; the dashed blue line indicates the number of people identified by the model as being at high risk for different probability thresholds and actually having an outcome event).

We developed a diagnostic nomogram for OA based on the expression of Hub OA-ARDEGs in order to obtain a more clinically applicable diagnostic model for OA. By constructing clinical calibration curves (**Figure 8B**), clinical decision curves (**Figure 8C**), and Clinical impact curve (**Figure 8D**) for the model, it is clear that the model has a high predictive power for OA. The scores of each gene expressed in the nomogram accurately predict the risk of OA disease (**Figure 8A**). The Hub OA-ARDEGs expression information and nomoscores of all samples are detailed in the **Supplementary Material: Table S12**.

### 3.9 Hub OA-ARDEGs expression and diagnostic value

**Figure 9:**
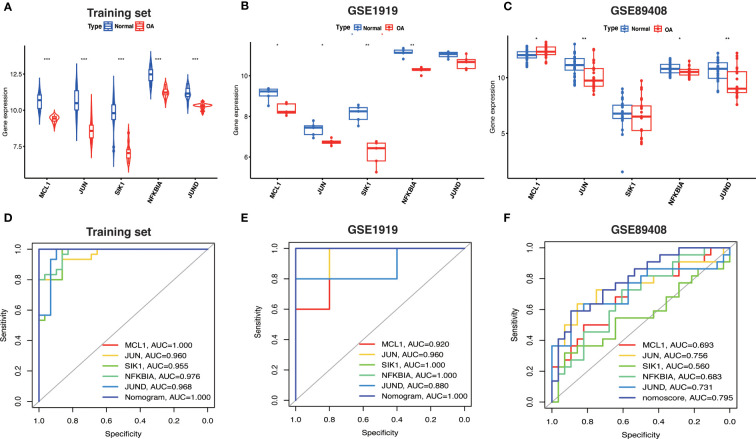
Hub OA-ARDEGs expression difference and ROC curve. **(A)** Violin plot of Hub OA-ARDEGs expression in normal and OA synovial tissue in the training set, *P<0.05; **P<0.01; ***P<0.001. **(B, C)** Box plot of Hub OA-ARDEGs expression in normal and OA synovial tissue in GSE1919 and GSE89408 validation set. **(D)** ROC curve analysis of Hub OA-ARDEGs and nomogram in the training set, *P<0.05; **P<0.01; ***P<0.001. **(E, F)** ROC curve analysis of Hub OA-ARDEGs and nomogram in the GSE1919 and GSE89408 validation set.

Based on the expression levels of Hub OA-ARDEGs in the training set and validation set, we found that Hub OA-ARDEGs were significantly downregulated in all OA synovial samples (**Figure 9A–C**). ROC curve analysis showed that the 5 Hub OA-ARDEGs and nomogram had high diagnostic values for OA in the training set. MCL1 and nomogram had the highest diagnostic value (AUC=1.000), and the other genes had the following diagnostic values: JUN (AUC=0.960), SIK1 (AUC=0.955), NFKBIA (AUC=0.976) and JUND (AUC=0.968) (**Figure 9D**). **Figures 9E, F** displays the ROC analysis results for the external validation set GSE1919 and GSE89408. The AUC for all five Hub OA-ARDEGs and nomogram in the validation set were greater than 0.5. As a result, the five Hub OA-ARDEGs can be used as reliable biomarkers for the diagnosis of OA and have a high diagnostic value.

### 3.10 qRT-PCR

**Figure 10:**
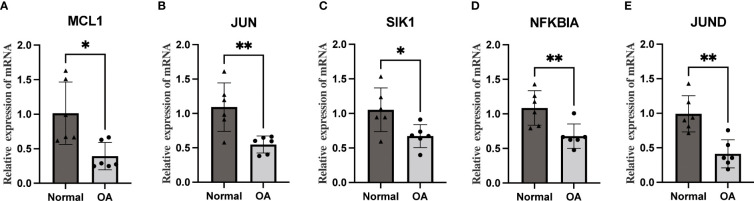
The qRT-PCR method was used to detect the mRNA expression levels of five Hub OA-ARDEGs. **(A-E)** MCL1, JUN, SIK1, NFKBIA, and JUND were significantly downregulated. *P<0.05, **P<0.01.

We performed qRT-PCR on total mRNA from synovial membranes of six patients with meniscal injuries and six patients with OA to further validate the mRNA expression levels of Hub OA-ARDEGs. The results showed that all five Hub OA-ARDEGs were significantly downregulated in the OA synovial samples (p-value less than 0.05), consistent with the expression in the training set (**Figures 10A–E**).

### 3.11 Construction of the miRNA-TF-mRNA regulatory network

**Figure 11:**
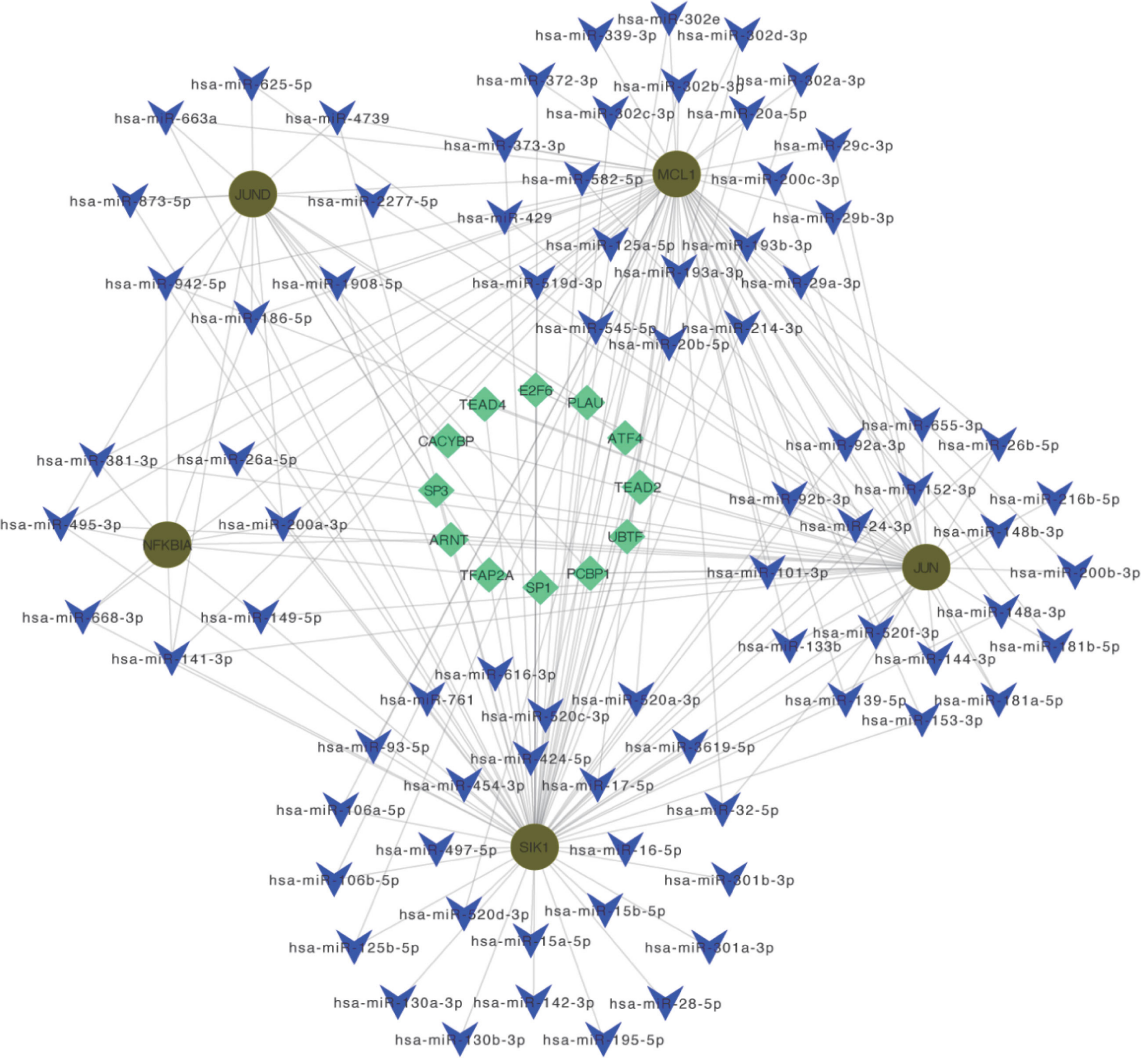
miRNA-TF-mRNA regulatory network. Diagram of miRNA-TF-mRNA regulatory network, where red circles represent genes; blue V-shapes represent predicted miRNAs; green diamonds represent TF.

By predicting miRNA and TF on Hub OA-ARDEGs, we used Cytoscape (3.7.1) to visualize the regulatory network, which contained 80 miRNAs, 12 transcription factors, and five genes, and obtained a total of 196 miRNA-TF-mRNA regulatory relationships (**Figure 11**). Details of miRNA-TF-mRNA regulatory networks were shown in **Supplementary Material: Table S13**.

### 3.12 Immune infiltration analysis

**Figure 12:**
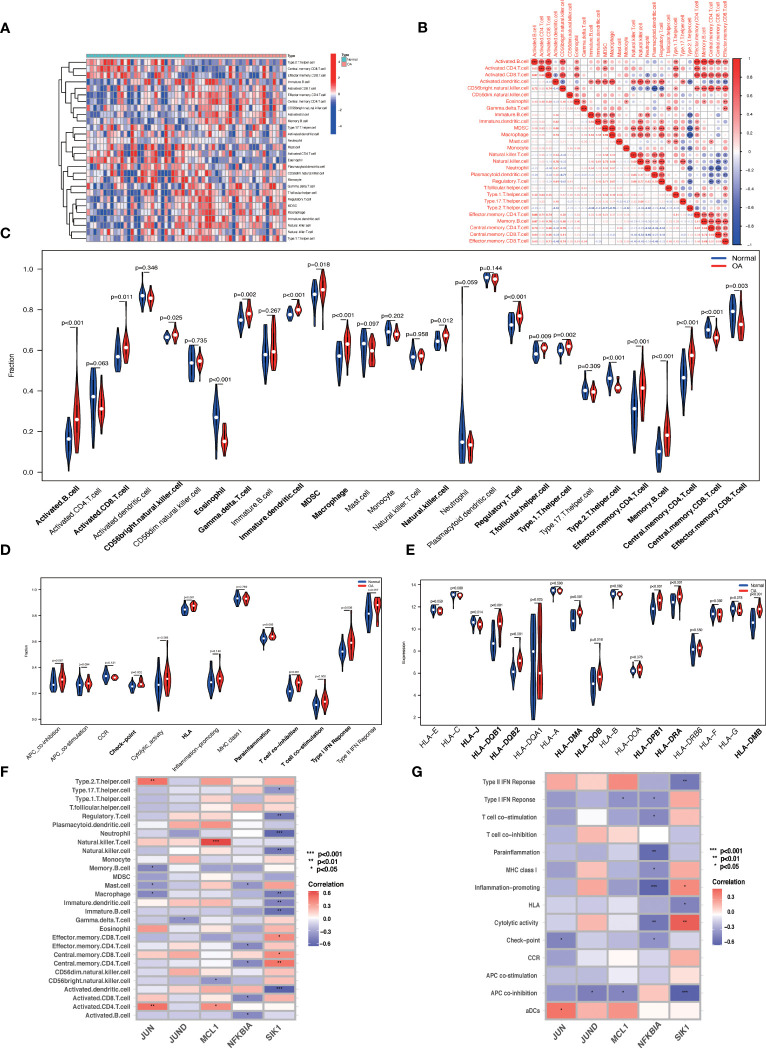
ssGSEA immune infiltration. **(A)** Heat map of the differences in the distribution of 28 immune cells in each sample. **(B)** Correlation analysis between 28 immune cell species. **(C, D)** Violin plots of differences in the infiltration of 28 immune cells and 13 immune functions in normal synovial and OA synovial samples. **(E)** Box plot of HLA gene expression differences in normal and OA synovial samples. **(F, G)** Correlation analysis of **Hub OA-ARDEGs** with 28 immune cells and 13 immune functions, *P<0.05, **P<0.01, ***P<0.001, ****P<0.0001.

We used the ssGSEA algorithm to discover that the infiltration levels of Activated B cell, Immature dendritic cell, Macrophage, Regulatory T cell, Central memory CD4 T cell, Memory B cell, and Effector memory CD4 T cell were significantly increased in OA samples. In contrast, the infiltration of Eosinophil, Type 2 T helper cell, and Central memory CD8 T cells in OA samples was significantly reduced (**Figures 12A, C**). The Spearman's correlation was applied to analyze the interaction between immune cells. The results revealed there are significant correlations between most immune cells, e.g. Macrophage and MDSC were significantly positively correlated (r = 0.89) (**Figure 12B**). Checkpoint, HLA, parainflammation, T cell co-inhibition, T cell co-stimulation, Type I IFN Response, and other immune functions were significantly activated in OA samples (**Figure 12D**). HLA gene expression levels were higher in OA samples, including HLA-DMA and HLA-DRA (**Figure 12E**), which indicates that the immune response plays an essential role in the development of OA. JUND, JUN, MCL1, NFKNIA, and SIK1 correlated well with a variety of immune cells and immune functions (**Figures 12F, G**). For example, JUN was positively correlated with Activated CD4 T cells, Type 2 T helper cells, and aDCs, and negatively correlated with Macrophage, Mast cells, Memory B cells, and Check-point. JUND was negatively correlated with Gamma delta T cells and APC co-inhibition. MCL1 was positively correlated with Natural killer T cell, Activated CD4 T cell, and negatively correlated with CD56 bright natural killer cell and APC co-inhibition. NFKBIA was negatively correlated with Activated B cell, Activated CD8 T cell, etc. SIK1 was positively correlated with Central memory CD4 T cell, Central memory CD8 T cell, and Cytolytic activity, and negatively correlated with Neutrophil, Regulatory T cell, Natural killer cell, Macrophage, APC co-inhibition, Type II IFN Response, and HLA. Data of results were shown in the **Supplementary Material: Table S14, 15**.

## 4 Discussion

OA is a chronic disease characterized by pain, cartilage loss, and joint inflammation, with aging playing a significant role in its progression. To date, OA cartilage has been extensively studied in terms of aging. Oxidative stress, for example, causes chondrocyte senescence by increasing p53 and p21 expression and activating the p38 MAPK and PI3K/Akt signaling pathways (28). Mechanical stress causes chondrocyte senescence and accelerates cartilage catabolism by downregulating FBXW7 (29). Sirt6 slows chondrocyte aging by inhibiting IL-15/JAK3/STAT5 signaling (30). However, a growing body of research has recently recognized that synovial aging plays an important role in OA, but the exact role and mechanisms remain unknown (31). As a result, the purpose of this study was to identify potential biomarkers of aging in OA and to investigate the role and mechanisms of Aging-related genes and immune infiltration in OA synovial tissues, thereby providing new directions and ideas for potential mechanisms and early OA diagnosis.

In normal and OA synovial samples, we discovered 87 ARDEGs. According to the GO and KEGG enrichment analysis results, ARDEGs are primarily involved in response to extracellular stimulus, response to nutrient levels, and HIF-1 signaling pathway, FoxO signaling pathway, PI3K-Akt signaling pathway, and MAPK signaling pathway, which is consistent with previous studies (32–35). It has been proposed that ARDEGs may be involved in inflammatory regulation, cellular stress response, cell cycle regulation, transcriptional regulation, and other mechanisms that promote OA progression. The results of the GSVA enrichment analysis revealed that TNFA signaling *via* the NFKB pathway, apoptosis, MAPK signaling pathway, and the P53 pathway were all significantly upregulated in OA. This is consistent with the functional enrichment of ARDEGs in OA. Using WGCNA analysis and three machine learning screenings, we were able to identify five Hub OA-ARDEGs (MCL1, SIK1, JUND, NFKBIA, JUN). Our findings suggested that Hub OA-ARDEGs had an excellent diagnostic ability to predict OA and were significantly downregulated in synovial samples from OA patients.

MCL1 (MCL1 Apoptosis Regulator, BCL2 Family Member) is an anti-apoptotic member of the BCL-2 family of proteins, which is involved in the regulation of apoptosis, cellular senescence, and inflammation and is essential for the maintenance of cell survival and viability (36). MCL1 expression is significantly downregulated in OA and senescent chondrocytes, and miR-34a-targeted inhibition of MCL1 can induce chondrocyte apoptosis as well as promote inflammatory response and ECM degradation (37). RHEB overexpression can, surprisingly, upregulate MCL1 to alleviate chondrocyte senescence and oxidative stress, which may be related to MCL1 inhibiting ROS production and P27 expression (38). MCL1 downregulation in OA synovial tissue and a positive correlation with natural killer cells suggest that MCL1 downregulation may be involved in synovial senescence and synovitis. However, more experimental support is required. SIK1 (Salt Inducible Kinase 1) is a member of the AMPK kinase subfamily that regulates cell cycle, gluconeogenesis, and lipogenesis (39). SIK1 has been shown in studies to inhibit TLR4-induced NF-B activation and reduce the expression of pro-inflammatory cytokines (40). However, SIK1 has not been reported in the OA literature. NFKBIA (NFKB Inhibitor Alpha) is an NFKB inhibitor that reduces the inflammatory response by inhibiting the activity of the dimeric NF-kappa-B/REL complex. Multiple studies have shown that abnormal NF-B activation is associated with chondrocyte catabolism, chondrocyte senescence, and synovial inflammation, and NF-B inhibition may be a potential therapeutic target for the treatment of OA (41, 42). Interestingly, the number of chondrocytes activated by NF-B signaling was significantly increased in aged articular cartilage, and activation of IKK-NF-B signaling in chondrocytes accelerated the occurrence of age-related joint tissue degeneration (43). Overexpression of NFKBIA in OA synovial fibroblasts effectively inhibits the expression of destructive enzymes such as MMP-1, MMP-3, and MMP-13, as well as ADAMTS4, reducing inflammation (44). These findings support our hypothesis that NFKBIA downregulation is important in promoting OA. JUND (JUND Proto-Oncogene, AP-1 Transcription Factor Subunit) is a functional component of the AP1 transcription factor complex, which is involved in cell proliferation, differentiation, and senescence, primarily through the regulation of oxidative stress levels (45, 46). JUND has been shown to protect cells from p53-dependent senescence and apoptosis, making it an appealing molecular target for preventing or delaying age-related cardiovascular disease (47, 48). In osteoarthritis, JUND transcriptional activation acts on the LncRNA LOXL1-AS1 to promote chondrocyte proliferation and inflammation, leading to osteoarthritis progression (49). The precise role of JUND in OA has yet to be determined. JUN (Jun Proto-Oncogene, AP-1 Transcription Factor Subunit) is a nuclear transcription-activating protein l family member involved in a variety of physiological processes such as cell cycle progression, differentiation, and apoptosis (50). JUN was discovered to slow aging by suppressing p53 gene transcription, whereas JUN knockout mouse embryonic fibroblasts (MEF) exhibit severe proliferation defects and early senescence (51). JUN binds to BATF to form a complex that regulates the expression of anabolic and catabolic genes in chondrocytes, which is critical in the destruction of OA cartilage (52). JUN overexpression has also been linked to reduced SOX9 transcriptional activity and type II collagen expression in chondrocytes (53). We discovered significant JUN downregulation in OA synovium, but it is unclear whether this promotes synovial aging and inflammation. More research into the specific role and mechanisms of Hub OA-ARDEGs in OA will hopefully lead to an emerging target for OA treatment.

Long-term low-level chronic inflammation and innate and adaptive immune system activation play critical roles in all aspects of OA pathogenesis (54, 55). Using ssGSEA analysis, we discovered that macrophages, natural killer cells, regulatory T cells, activated CD8 T cells, central memory CD4 T cells, effector memory CD4 T cells, activated B cells, and memory B cells were significantly infiltrated in OA. Checkpoint, HLA, parainflammation, and other immune functions were significantly activated in OA samples. Macrophages, the primary innate immune cells in the OA synovium, are present in 76% of OA patients’ knee joints and are significantly associated with knee pain, OA radiographic severity, and osteophytes (56). Through the secretion of inflammatory, growth factors and MMPs, activated macrophages cause chondrocyte senescence and apoptosis (57). NK cells, as one of the innate immune cells, play an important role in monitoring and killing senescent cells. In the liver, NK cells use perforin granule exocytosis to target the clearance of senescent hepatic stellate cells (58). The recruitment and activation of NK cells is thought to accelerate the progression of OA in a mouse model of osteoarthritis with cartilage damage (59). Similarly, the adaptive immune system unquestionably plays a role in OA. Numerous studies have found a significant infiltration of T cells in OA synovial and synovial fluid, second only to macrophages in accounting for 25% of inflammatory cells (60). CD8+ T-cell-induced tissue inhibitor of metalloprotein-1 (TIMP1) expression aggravates osteoarthritis (61). The number of synovial CD4+ T lymphocytes correlates with a visual analog pain scale, promotes Th1 cell polarization, and increases the release of immunomodulatory cytokines, accelerating the progression of OA (62, 63). B cells and plasma cells have been found in synovial tissue from OA patients. However, no studies have found a direct link between B cell infiltration and OA progression or severity, and further experimental confirmation of their specific role is required (64). We used Spearman correlation analysis to show that the five Hub OA-ARDEGs have a reasonable correlation with immune cells and functions, implying that synovial immune inflammation may collaborate with aging to contribute to the occurrence and progression of OA.

However, there are limitations to this study:

1. The transcriptomic data for this study were sourced from publicly available databases, which may limit access to more clinically relevant information. The variability of patient populations and clinical characteristics cannot be ruled out as a possible influence on the study’s findings.
2. The limited sample size had a potential impact on the accuracy of the results, emphasizing the need for a larger sample size and a prospective study design to validate and reinforce the model’s findings.
3. We conducted an extensive exploration and analysis of databases and supplemented our findings with limited experimental validations. However, it is important to note that our study would benefit from additional experimental support to further strengthen the conclusions.

## 5 Conclusion

In conclusion, this study preliminarily investigated the potential mechanisms of aging-related genes in OA synovial tissue, which revealed that synovial aging could be closely linked to immune inflammation. In addition, five Hub OA-ARDEGs have excellent diagnostic capabilities for OA and may be novel targets for the diagnosis and treatment of OA. However, additional experimental studies are required to support our findings.

## Data availability statement

The original contributions presented in the study are included in the article/**Supplementary Material**. Further inquiries can be directed to the corresponding authors.

## Ethics statement

The studies involving human participants were reviewed and approved by Guangzhou Red Cross Hospital of Jinan University (Ethics number 2023-001-01). The patients/participants provided their written informed consent to participate in this study.

## Author contributions

QM and LW directed the research and revised the manuscript. JZ and JH conceived the idea and wrote the manuscript, they have contributed equally to this work and share first authorship. ZL and QS are responsible for analyzing and processing the data. ZY processed and modified the image. All authors contributed to the article and approved the submitted version.

## References

[1] Prieto-Alhambra D Judge A Javaid MK Cooper C Diez-Perez A Arden NK. Incidence and risk factors for clinically diagnosed knee, hip and hand osteoarthritis: influences of age, gender and osteoarthritis affecting other joints. Ann Rheum Dis (2014) 73(9):1659–64. doi: 10.1136/annrheumdis-2013-203355 PMC3875433 23744977

[2] James SL Abate D Abate KH Abay SM Abbafati C Abbasi N.Global, regional, and national incidence, prevalence, and years lived with disability for 354 diseases and injuries for 195 countries and territories, 1990-2017: a systematic analysis for the global burden of disease study 2017. Lancet (2018) 392(10159):1789–858. doi: 10.1016/s0140-6736(18)32279-7 PMC6227754 30496104

[3] Glyn-Jones S Palmer AJ Agricola R Price AJ Vincent TL Weinans H. Osteoarthritis. Lancet (2015) 386(9991):376–87. doi: 10.1016/s0140-6736(14)60802-3 25748615

[4] Kirkwood TB. Understanding the odd science of aging. Cell (2005) 120(4):437–47. doi: 10.1016/j.cell.2005.01.027 15734677

[5] Zhu Y Armstrong JL Tchkonia T Kirkland JL. Cellular senescence and the senescent secretory phenotype in age-related chronic diseases. Curr Opin Clin Nutr Metab Care (2014) 17(4):324–8. doi: 10.1097/mco.0000000000000065 24848532

[6] MacNee W Rabinovich RA Choudhury G. Ageing and the border between health and disease. Eur Respir J (2014) 44(5):1332–52. doi: 10.1183/09031936.00134014 25323246

[7] Muñoz-Espín D Serrano M. Cellular senescence: from physiology to pathology. Nat Rev Mol Cell Biol (2014) 15(7):482–96. doi: 10.1038/nrm3823 24954210

[8] Rodier F Coppé JP Patil CK Hoeijmakers WA Muñoz DP Raza SR. Persistent DNA damage signalling triggers senescence-associated inflammatory cytokine secretion. Nat Cell Biol (2009) 11(8):973–9. doi: 10.1038/ncb1909 PMC2743561 19597488

[9] Greene MA Loeser RF. Aging-related inflammation in osteoarthritis. Osteoarthritis Cartilage (2015) 23(11):1966–71. doi: 10.1016/j.joca.2015.01.008 PMC4630808 26521742

[10] Del Rey MJ Valín Á Usategui A Ergueta S Martín E Municio C. Senescent synovial fibroblasts accumulate prematurely in rheumatoid arthritis tissues and display an enhanced inflammatory phenotype. Immun Ageing (2019) 16:29. doi: 10.1186/s12979-019-0169-4 31708994 PMC6833299

[11] Xu M Bradley EW Weivoda MM Hwang SM Pirtskhalava T Decklever T. Transplanted senescent cells induce an osteoarthritis-like condition in mice. J Gerontol A Biol Sci Med Sci (2017) 72(6):780–5. doi: 10.1093/gerona/glw154 PMC5861939 27516624

[12] Jeon OH Kim C Laberge RM Demaria M Rathod S Vasserot AP. Local clearance of senescent cells attenuates the development of post-traumatic osteoarthritis and creates a pro-regenerative environment. Nat Med (2017) 23(6):775–81. doi: 10.1038/nm.4324 PMC5785239 28436958

[13] Zhao X Zhang L Wang J Zhang M Song Z Ni B. Identification of key biomarkers and immune infiltration in systemic lupus erythematosus by integrated bioinformatics analysis. J Transl Med (2021) 19(1):35. doi: 10.1186/s12967-020-02698-x 33468161 PMC7814551

[14] Waljee AK Weinheimer-Haus EM Abubakar A Ngugi AK Siwo GH Kwakye G. Artificial intelligence and machine learning for early detection and diagnosis of colorectal cancer in Sub-Saharan Africa. Gut (2022) 71(7):1259–65. doi: 10.1136/gutjnl-2022-327211 PMC9177787 35418482

[15] Leek JT Johnson WE Parker HS Jaffe AE Storey JD. The sva package for removing batch effects and other unwanted variation in high-throughput experiments. Bioinformatics (2012) 28(6):882–3. doi: 10.1093/bioinformatics/bts034 PMC3307112 22257669

[16] Tacutu R Thornton D Johnson E Budovsky A Barardo D Craig T. Human ageing genomic resources: new and updated databases. Nucleic Acids Res (2018) 46(D1):D1083–d90. doi: 10.1093/nar/gkx1042 PMC5753192 29121237

[17] Ritchie ME Phipson B Wu D Hu Y Law CW Shi W. Limma powers differential expression analyses for rna-sequencing and microarray studies. Nucleic Acids Res (2015) 43(7):e47. doi: 10.1093/nar/gkv007 25605792 PMC4402510

[18] Szklarczyk D Gable AL Lyon D Junge A Wyder S Huerta-Cepas J. String V11: protein-protein association networks with increased coverage, supporting functional discovery in genome-wide experimental datasets. Nucleic Acids Res (2019) 47(D1):D607–d13. doi: 10.1093/nar/gky1131 PMC6323986 30476243

[19] Hänzelmann S Castelo R Guinney J. Gsva: gene set variation analysis for microarray and rna-seq data. BMC Bioinf (2013) 14:7. doi: 10.1186/1471-2105-14-7 PMC3618321 23323831

[20] Langfelder P Horvath S. Wgcna: an r package for weighted correlation network analysis. BMC Bioinf (2008) 9:559. doi: 10.1186/1471-2105-9-559 PMC2631488 19114008

[21] Frost HR Amos CI. Gene set selection *Via* lasso penalized regression (Slpr). Nucleic Acids Res (2017) 45(12):e114. doi: 10.1093/nar/gkx291 28472344 PMC5499546

[22] Nedaie A Najafi AA. Support vector machine with dirichlet feature mapping. Neural Netw (2018) 98:87–101. doi: 10.1016/j.neunet.2017.11.006 29223012

[23] Yuan Y Fu M Li N Ye M. Identification of immune infiltration and cuproptosis-related subgroups in crohn’s disease. Front Immunol (2022) 13:1074271. doi: 10.3389/fimmu.2022.1074271 36466876 PMC9713932

[24] Chou CH Shrestha S Yang CD Chang NW Lin YL Liao KW. Mirtarbase update 2018: a resource for experimentally validated microrna-target interactions. Nucleic Acids Res (2018) 46(D1):D296–d302. doi: 10.1093/nar/gkx1067 29126174 PMC5753222

[25] Yang JH Li JH Shao P Zhou H Chen YQ Qu LH. Starbase: a database for exploring microrna-mrna interaction maps from argonaute clip-seq and degradome-seq data. Nucleic Acids Res (2011) 39(Database issue):D202–9. doi: 10.1093/nar/gkq1056 PMC3013664 21037263

[26] Agarwal V Bell GW Nam JW Bartel DP. Predicting effective microrna target sites in mammalian mrnas. Elife (2015) 4:e05005. doi: 10.7554/eLife.05005 26267216 PMC4532895

[27] Barbie DA Tamayo P Boehm JS Kim SY Moody SE Dunn IF. Systematic rna interference reveals that oncogenic kras-driven cancers require Tbk1. Nature (2009) 462(7269):108–12. doi: 10.1038/nature08460 PMC2783335 19847166

[28] Jeon OH David N Campisi J Elisseeff JH. Senescent cells and osteoarthritis: a painful connection. J Clin Invest (2018) 128(4):1229–37. doi: 10.1172/jci95147 PMC5873863 29608139

[29] Zhang H Shao Y Yao Z Liu L Zhang H Yin J. Mechanical overloading promotes chondrocyte senescence and osteoarthritis development through downregulating Fbxw7. Ann Rheum Dis (2022) 81(5):676–86. doi: 10.1136/annrheumdis-2021-221513 35058228

[30] Ji ML Jiang H Li Z Geng R Hu JZ Lin YC. Sirt6 attenuates chondrocyte senescence and osteoarthritis progression. Nat Commun (2022) 13(1):7658. doi: 10.1038/s41467-022-35424-w 36496445 PMC9741608

[31] Xie J Wang Y Lu L Liu L Yu X Pei F. Cellular senescence in knee osteoarthritis: molecular mechanisms and therapeutic implications. Ageing Res Rev (2021) 70:101413. doi: 10.1016/j.arr.2021.101413 34298194

[32] Zeng CY Wang XF Hua FZ. Hif-1α in osteoarthritis: from pathogenesis to therapeutic implications. Front Pharmacol (2022) 13:927126. doi: 10.3389/fphar.2022.927126 35865944 PMC9294386

[33] Sun K Luo J Guo J Yao X Jing X Guo F. The Pi3k/Akt/Mtor signaling pathway in osteoarthritis: a narrative review. Osteoarthritis Cartilage (2020) 28(4):400–9. doi: 10.1016/j.joca.2020.02.027 32081707

[34] Lee KI Choi S Matsuzaki T Alvarez-Garcia O Olmer M Grogan SP. Foxo1 and Foxo3 transcription factors have unique functions in meniscus development and homeostasis during aging and osteoarthritis. Proc Natl Acad Sci U.S.A. (2020) 117(6):3135–43. doi: 10.1073/pnas.1918673117 PMC7022148 31980519

[35] Li Z Dai A Yang M Chen S Deng Z Li L. P38mapk signaling pathway in osteoarthritis: pathological and therapeutic aspects. J Inflammation Res (2022) 15:723–34. doi: 10.2147/jir.S348491 PMC8820459 35140502

[36] Widden H Placzek WJ. The multiple mechanisms of Mcl1 in the regulation of cell fate. Commun Biol (2021) 4(1):1029. doi: 10.1038/s42003-021-02564-6 34475520 PMC8413315

[37] Xiong S Zhao Y Xu T. DNA Methyltransferase 3 beta mediates the methylation of the microrna-34a promoter and enhances chondrocyte viability in osteoarthritis. Bioengineered (2021) 12(2):11138–55. doi: 10.1080/21655979.2021.2005308 PMC8810119 34783292

[38] Ashraf S Ahn J Cha BH Kim JS Han I Park H. Rheb: a potential regulator of chondrocyte phenotype for cartilage tissue regeneration. J Tissue Eng Regener Med (2017) 11(9):2503–15. doi: 10.1002/term.2148 27061379

[39] Sakamoto K Bultot L Göransson O. The salt-inducible kinases: emerging metabolic regulators. Trends Endocrinol Metab (2018) 29(12):827–40. doi: 10.1016/j.tem.2018.09.007 30385008

[40] Yong Kim S Jeong S Chah KH Jung E Baek KH Kim ST. Salt-inducible kinases 1 and 3 negatively regulate toll-like receptor 4-mediated signal. Mol Endocrinol (2013) 27(11):1958–68. doi: 10.1210/me.2013-1240 PMC5427829 24061540

[41] Chien Y Scuoppo C Wang X Fang X Balgley B Bolden JE. Control of the senescence-associated secretory phenotype by nf-Kb promotes senescence and enhances chemosensitivity. Genes Dev (2011) 25(20):2125–36. doi: 10.1101/gad.17276711 PMC3205583 21979375

[42] Choi MC Jo J Park J Kang HK Park Y. Nf-Kb signaling pathways in osteoarthritic cartilage destruction. Cells (2019) 8(7):734. doi: 10.3390/cells8070734 PMC6678954 31319599

[43] Catheline SE Bell RD Oluoch LS James MN Escalera-Rivera K Maynard RD. Ikkβ-Nf-Kb signaling in adult chondrocytes promotes the onset of age-related osteoarthritis in mice. Sci Signal (2021) 14(701):eabf3535. doi: 10.1126/scisignal.abf3535 34546791 PMC8734558

[44] Bondeson J Lauder S Wainwright S Amos N Evans A Hughes C. Adenoviral gene transfer of the endogenous inhibitor ikappabalpha into human osteoarthritis synovial fibroblasts demonstrates that several matrix metalloproteinases and aggrecanases are nuclear factor-Kappab-Dependent. J Rheumatol (2007) 34(3):523–33. 17295438

[45] Hernandez JM Floyd DH Weilbaecher KN Green PL Boris-Lawrie K. Multiple facets of jund gene expression are atypical among ap-1 family members. Oncogene (2008) 27(35):4757–67. doi: 10.1038/onc.2008.120 PMC2726657 18427548

[46] Gerald D Berra E Frapart YM Chan DA Giaccia AJ Mansuy D. Jund reduces tumor angiogenesis by protecting cells from oxidative stress. Cell (2004) 118(6):781–94. doi: 10.1016/j.cell.2004.08.025 15369676

[47] Short JD Pfarr CM. Translational regulation of the jund messenger rna. J Biol Chem (2002) 277(36):32697–705. doi: 10.1074/jbc.M204553200 12105216

[48] Costantino S Paneni F Cosentino F. Ageing, metabolism and cardiovascular disease. J Physiol (2016) 594(8):2061–73. doi: 10.1113/jp270538 PMC4933114 26391109

[49] Chen K Fang H Xu N. Lncrna Loxl1-As1 is transcriptionally activated by jund and contributes to osteoarthritis progression *Via* targeting the mir-423-5p/Kdm5c axis. Life Sci (2020) 258:118095. doi: 10.1016/j.lfs.2020.118095 32679142

[50] Meng Q Xia Y. C-jun, at the crossroad of the signaling network. . Protein Cell (2011) 2(11):889–98. doi: 10.1007/s13238-011-1113-3 PMC4875184 22180088

[51] Meixner A Karreth F Kenner L Penninger JM Wagner EF. Jun and jund-dependent functions in cell proliferation and stress response. Cell Death Differ (2010) 17(9):1409–19. doi: 10.1038/cdd.2010.22 20300111

[52] Rhee J Park SH Kim SK Kim JH Ha CW Chun CH. Inhibition of Batf/Jun transcriptional activity protects against osteoarthritic cartilage destruction. Ann Rheum Dis (2017) 76(2):427–34. doi: 10.1136/annrheumdis-2015-208953 PMC5284350 27147707

[53] Hwang SG Yu SS Poo H Chun JS. C-Jun/Activator protein-1 mediates interleukin-1beta-Induced dedifferentiation but not cyclooxygenase-2 expression in articular chondrocytes. . J Biol Chem (2005) 280(33):29780–7. doi: 10.1074/jbc.M411793200 15961395

[54] Robinson WH Lepus CM Wang Q Raghu H Mao R Lindstrom TM. Low-grade inflammation as a key mediator of the pathogenesis of osteoarthritis. Nat Rev Rheumatol (2016) 12(10):580–92. doi: 10.1038/nrrheum.2016.136 PMC5500215 27539668

[55] Liu-Bryan R Terkeltaub R. Emerging regulators of the inflammatory process in osteoarthritis. Nat Rev Rheumatol (2015) 11(1):35–44. doi: 10.1038/nrrheum.2014.162 25266449 PMC4374654

[56] Kraus VB McDaniel G Huebner JL Stabler TV Pieper CF Shipes SW. Direct *In vivo* evidence of activated macrophages in human osteoarthritis. Osteoarthritis Cartilage (2016) 24(9):1613–21. doi: 10.1016/j.joca.2016.04.010 PMC4992586 27084348

[57] Xie J Huang Z Yu X Zhou L Pei F. Clinical implications of macrophage dysfunction in the development of osteoarthritis of the knee. Cytokine Growth Factor Rev (2019) 46:36–44. doi: 10.1016/j.cytogfr.2019.03.004 30910350

[58] Burton DGA Stolzing A. Cellular senescence: immunosurveillance and future immunotherapy. Ageing Res Rev (2018) 43:17–25. doi: 10.1016/j.arr.2018.02.001 29427795

[59] Benigni G Dimitrova P Antonangeli F Sanseviero E Milanova V Blom A. Cxcr3/Cxcl10 axis regulates neutrophil-nk cell cross-talk determining the severity of experimental osteoarthritis. J Immunol (2017) 198(5):2115–24. doi: 10.4049/jimmunol.1601359 28108560

[60] Pessler F Chen LX Dai L Gomez-Vaquero C Diaz-Torne C Paessler ME. A histomorphometric analysis of synovial biopsies from individuals with gulf war veterans’ illness and joint pain compared to normal and osteoarthritis synovium. Clin Rheumatol (2008) 27(9):1127–34. doi: 10.1007/s10067-008-0878-0 18414968

[61] Hsieh JL Shiau AL Lee CH Yang SJ Lee BO Jou IM. Cd8+ T cell-induced expression of tissue inhibitor of metalloproteinses-1 exacerbated osteoarthritis. Int J Mol Sci (2013) 14(10):19951–70. doi: 10.3390/ijms141019951 PMC3821596 24108368

[62] Li YS Luo W Zhu SA Lei GH. T Cells in osteoarthritis: alterations and beyond. Front Immunol (2017) 8:356. doi: 10.3389/fimmu.2017.00356 28424692 PMC5371609

[63] Klein-Wieringa IR de Lange-Brokaar BJ Yusuf E Andersen SN Kwekkeboom JC Kroon HM. Inflammatory cells in patients with endstage knee osteoarthritis: a comparison between the synovium and the infrapatellar fat pad. J Rheumatol (2016) 43(4):771–8. doi: 10.3899/jrheum.151068 26980579

[64] Motta F Barone E Sica A Selmi C. Inflammaging and osteoarthritis. Clin Rev Allergy Immunol (2022) 64:222–238. doi: 10.1007/s12016-022-08941-1 35716253

