# Supplementary material for: Identification of aging-related biomarkers and immune infiltration characteristics in osteoarthritis based on bioinformatics analysis and machine learning
Source: Front Immunol. 2023 Jul 12;14:1168780. doi: 10.3389/fimmu.2023.1168780 (PMC10368975; doi:10.3389/fimmu.2023.1168780)
Supplement: Supplementary file 1 [file DataSheet_1.docx]

**
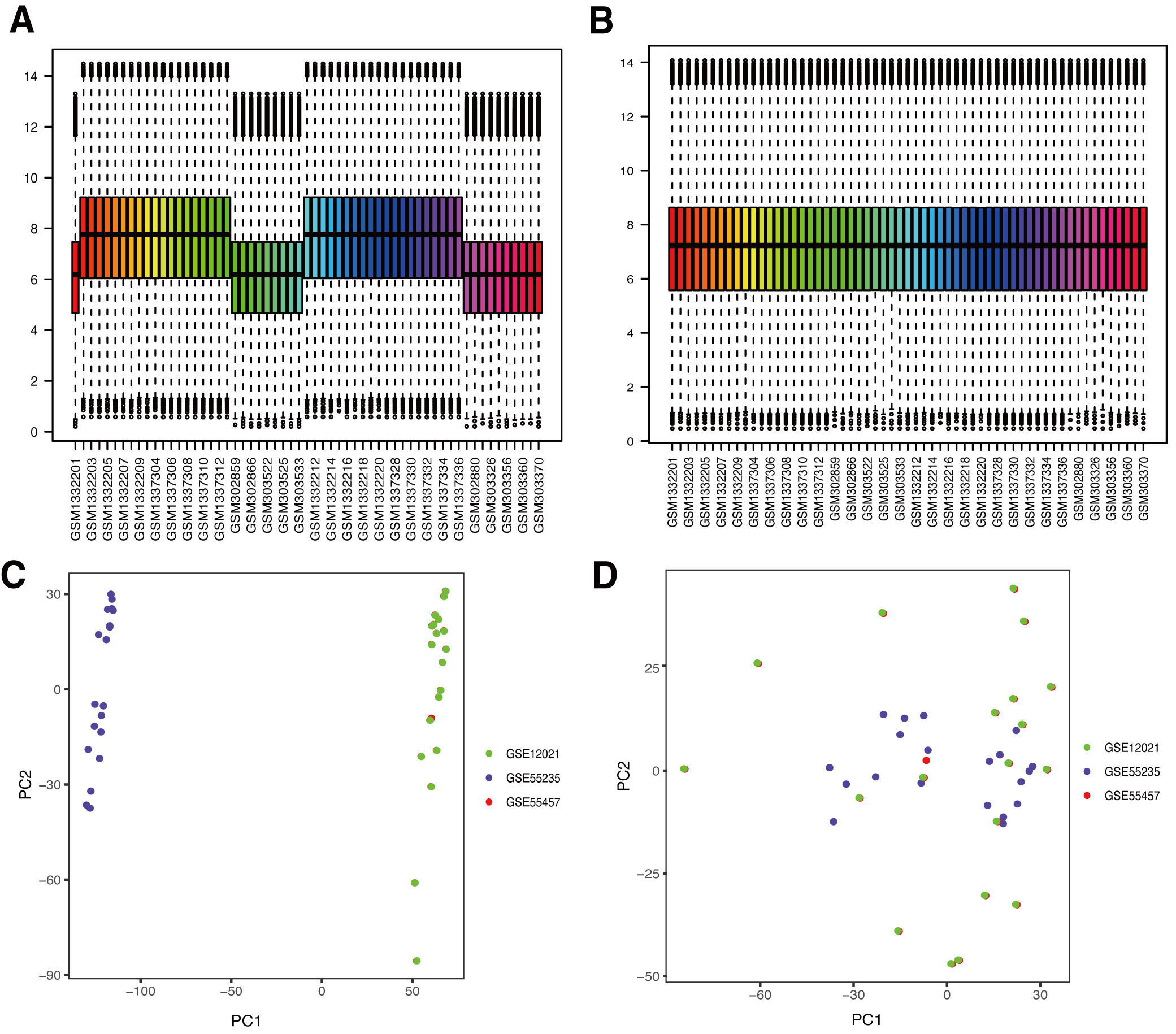
**

Figure 1

Data Processing. **(A, B)** Gene expression levels for each sample before and after normalization to remove batch effects. **(C, D)** Two-dimensional PCA clustering plot for each sample before and after normalization to remove batch effects.


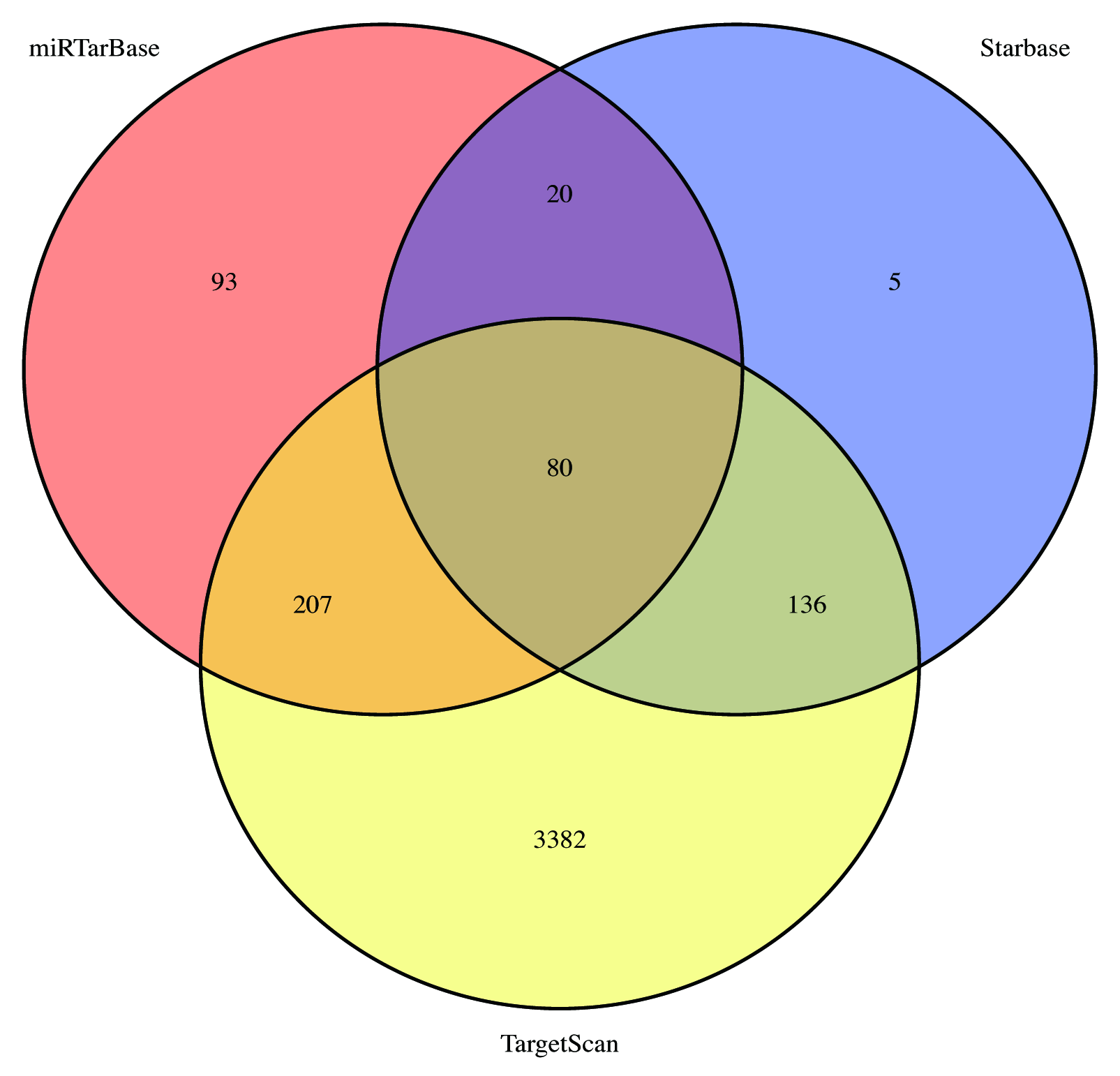


Figure 2

Three databases predicting miRNAs at the intersection of five Hub OA-ARDEGs.
